# Sustained Complete Response to Palbociclib in a Refractory Pediatric Sarcoma With *BCOR*-*CCNB3* Fusion and Germline *CDKN2B* Variant

**DOI:** 10.1200/PO.19.00258

**Published:** 2020-04-30

**Authors:** Timothy F. Tramontana, Mark S. Marshall, Amy E. Helvie, Morgan R. Schmitt, Jennifer Ivanovich, Jacquelyn L. Carter, Jamie L. Renbarger, Michael J. Ferguson

**Affiliations:** ^1^ Department of Genetics, Indiana University School of Medicine, Indianapolis, IN; ^2^ Department of Pediatrics, Indiana University School of Medicine, Indianapolis, IN

## Introduction

Genomic alterations in the Ewing sarcoma family of tumors (EFT) were discovered > 30 years ago with the identification of the reciprocal translocation, t(11;22)(q24;q12), otherwise known as EWS-FL1.^1,2^ In the time since, multiple other fusion partners with EWS have been identified that fit a similar Ewing sarcoma phenotype.^3,4^ When EWS fusions are not identified, tumors with histologic features of Ewing sarcoma have been labeled as primitive neuroectodermal tumors. In 2012, Pierron et al^5^ identified a subset of Ewing-like tumors harboring paracentric inversion on the short arm of chromosome X, resulting in the fusion of the *BCOR* and *CCNB3* genes.^5^ Since that discovery, several small case series have further elucidated the clinical, morphologic, and genomic differences that make this diagnosis distinct from other round cell sarcomas, most notably Ewing sarcoma.^6-8^

Though distinct from Ewing sarcoma, most *BCOR*-*CCNB3*–fused sarcomas (BCS) are treated with upfront compressed chemotherapy with vincristine, doxorubicin, cyclophosphamide, ifosfamide, and etoposide plus local control with surgery and/or radiation. BCS shares similar event-free and overall survival rates with the standard EWS-FLI1–fused Ewing sarcoma using this treatment strategy.^6-8^ Despite the growing knowledge base related to BCS, little is known about potential drug targets related to this disease entity, especially with regard to treatment of disease recurrence. We highlight the treatment of a young patient who had multiply-relapsed disease with the US Food and Drug Administration–approved cyclin-dependent kinase 4/6 (CDK4/6) inhibitor palbociclib; the tumor harbored a *BCOR*-*CCNB3* fusion and a germline variant in *CDKN2B*, and treatment resulted in a complete response and no evidence of disease 25 months into therapy.

## Case History

**FIG 1.**
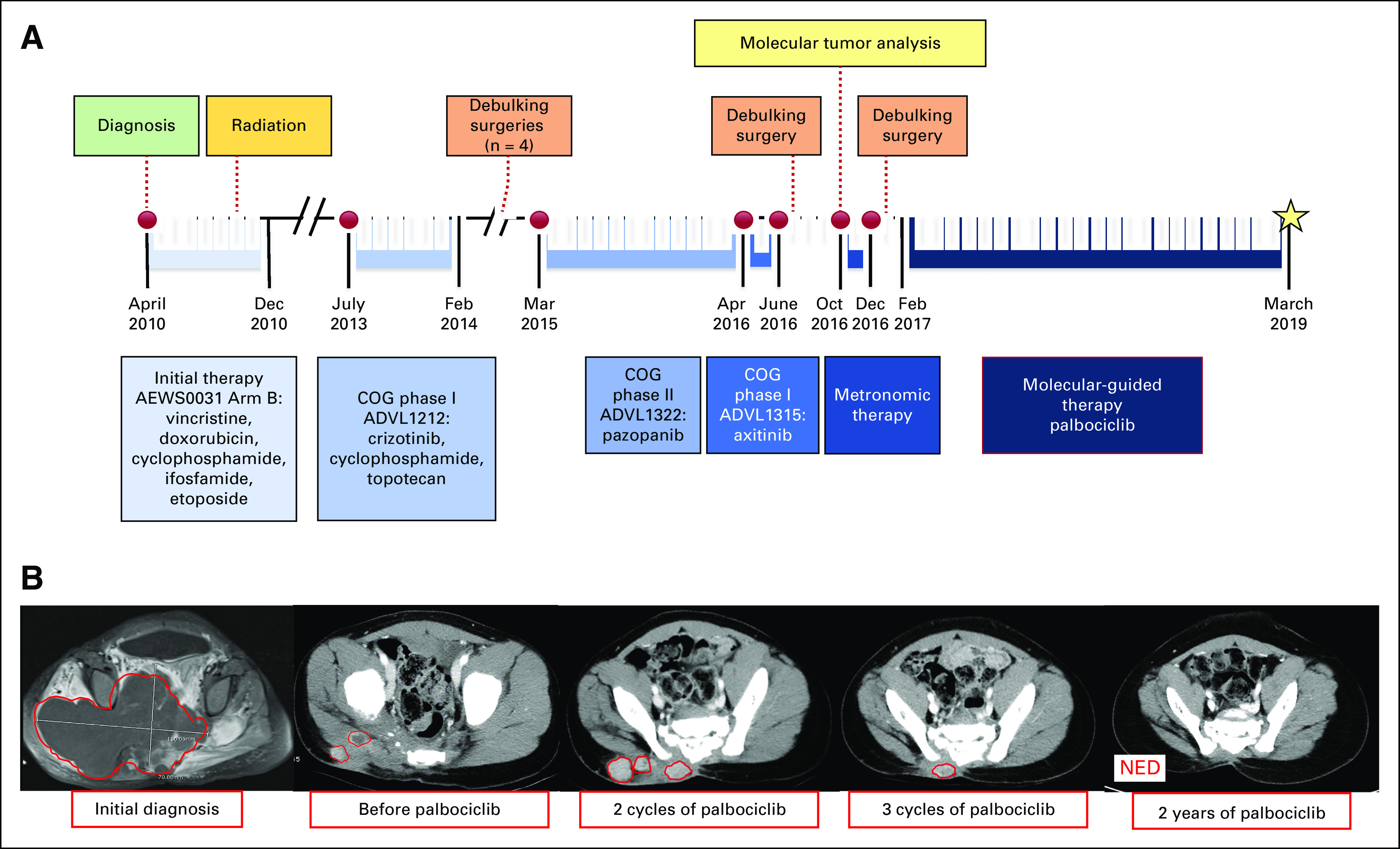
Patient timeline and response imaging. (A) The timeline of treatments and disease recurrences and progression in our patient. Red circles represent time of recurrence or progression. Hash marks represent prolonged period of time progressed through therapy (not to scale of remaining timeline). Patient is still continuing with palbociclib therapy after the most recent scans in March 2019. (B) Patient imaging displayed with initial diagnostic magnetic resonance imaging from 2010; subsequent relapse scans were done via computed tomography imaging. Red lines outline tumor boundaries. There was concern for progression after 2 cycles of palbociclib, but there was a 2-month lag between the “before palbociclib” scan and actually starting drug, so interval progression likely occurred in this timeframe. No new baseline scan was performed on the day palbociclib started. Each cycle of palbociclib was 28 days. NED, no evidence of disease.

Our male patient initially presented in 2010 at 1 year of age with a fixed mass on his back. Magnetic resonance imaging of the pelvis showed a large infiltrating presacral mass measuring 14 × 7.4 × 10.4 cm extending into the lower spinal canal, eroding the posterior right sacrum, and exerting a mass effect on both the rectum and bladder. A core needle biopsy was performed, which revealed a malignant, small, round, blue cell tumor along with small amounts of benign fibrofatty tissue and skeletal muscle. Tumor nuclei were round to oval with a fine-grained chromatin pattern and occasional small nucleoli or chromocenters. Immunohistochemical stains were positive for CD99, Fli1, and vimentin and were negative for NSE, synaptophysin, MYF4, GAF, CD45RB, and TdT—consistent with a primitive neuroectodermal tumor. No polymerase chain reaction–base fusion analysis or breakapart fluorescence in situ hybridization probe for *EWSR1* was performed at the time. Three generations of family history were negative for malignancies on either side of the family, including melanoma or pancreatic cancer. A staging computed tomography scan of the chest and a bone scan showed no evidence of metastatic disease. The patient started chemotherapy per Children’s Oncology Group protocol AEWS0031, regimen B2, with ifosfamide, etoposide, vincristine, doxorubicin, and cyclophosphamide. Gross total resection was not feasible at the time per neurosurgery, and the patient received 57.6 Gy of proton beam radiation in October 2010. The patient remained in remission for > 2 years but then developed multiple local recurrences without metastases from 2013 to 2017 and underwent numerous surgeries, along with multiple different early-phase Children’s Oncology Group therapeutic studies, as outlined in the timeline in Figure 1A. After the most recent recurrence in October 2016, the patient was referred to our Pediatric Cancer Precision Genomics Program. Because of the findings outlined here in the Results, we chose to start palbociclib in February 2017. This patient has no evidence of disease on imaging 25 months into therapy (Fig 1B) and has had only hematologic toxicity that was grade 2 or less.

## Results

**FIG 2.**
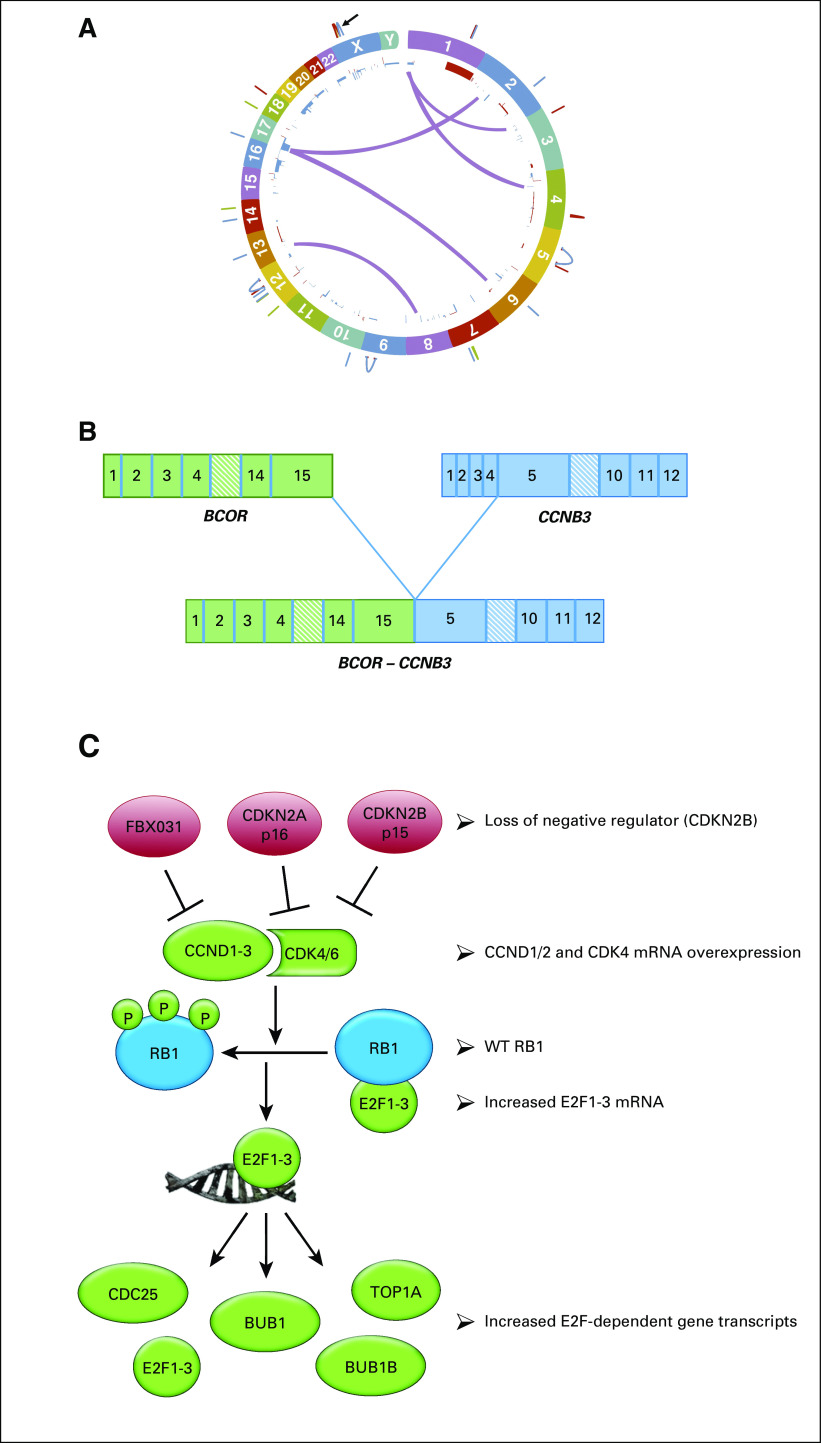
Transcriptome analysis reveals activation of the cyclin-dependent kinase 4/6 (CDK4/6)-RB pathway. (A) Circos plot of patient’s tumor genome. (B) Identification of BCOR-CCNB3 fusion. This graphic illustrates the fusion of BCOR exon 15 to CCNB3 exon 5. With the exception of the destruction box in CCNB3, all functional domains from each encoded protein remain intact in the BCOR-CCNB3 fusion protein. (C) The CDK4/6 pathway is a gene regulatory program controlled by multiple tiers of protein kinases and transcriptional regulators. Increases in cyclin-D or CDK4 or 6 protein can lead to phosphorylation of the RB1-E2F tumor suppressor complex. Upon phosphorylation of RB1, the E2F1-3 transcription factors are released from the complex and are able to bind to the promoters of target genes, driving activation of transcription. These target genes include E2F1-3 themselves, the *CDC25* genes, *TOP2A*, *BUB1*, and *BUB1B*. WT, wild type.

Whole-genome sequencing, RNA sequencing (RNA-Seq) analysis, germline exome sequencing, and protein evaluation were performed at the Clinical Laboratory Improvement Amendment (CLIA)–approved laboratory, NantOmics (Culver City, CA). Somatic DNA changes were determined by comparing the whole-genome DNA sequence from the tumor with the patient’s germline sequence at 33X coverage. The mutational burden of the tumor was relatively low at 75,046 somatic mutations, with only 88 somatic mutations mapping to protein coding regions (Circos plot in Fig 2A). The tumor harbored an in-frame fusion of the second base in the last codon of BCOR exon 15 (chrX:39,911,366) and the first base of CCNB3 exon 5 (chrX:50,051,505) (Fig 2B). Additionally, an undescribed somatic mutation in the *SMO* gene (*SMO* N476S) was identified in the tumor, and germline sequencing revealed a *CDKN2B* N41D missense variant, which was heterozygous in both the germline and tumor genomes of this patient. RNA-Seq was also performed by NantOmics, and mRNA transcripts were ranked by abundance, which could be associated with increased pathway activity and sensitivity to a targeted drug. Overexpression of relevant tumor-promoting pathways is displayed in Table 1 and Figure 2C.

**TABLE 1.**
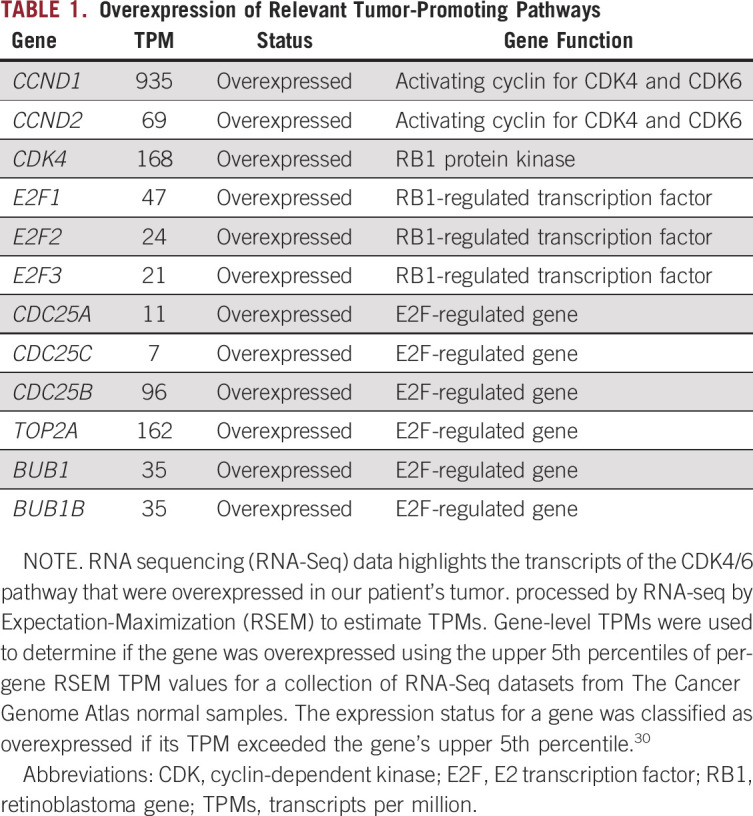
**Overexpression** **of Relevant Tumor-Promoting Pathways**

## Discussion

Germline and somatic whole-genome DNA sequencing combined with RNA sequencing was used with the goal of developing a treatment plan, and it surprisingly provided our team with a more defined diagnosis of a recurrent BCS. The specific intrachromosomal fusion between *BCOR* and *CCNB3* in our patient’s tumor is identical to previously described cases.^5,6^ The *BCOR* gene itself can fuse to a number of 3′ partner genes in round cell sarcomas or additionally have internal tandem duplications, which have been reported to drive similar transcriptional patterns in a variety of sarcomas.^5,8,9^ Similar to previous studies of mRNA transcripts in BCS,^5,8,9^ both *BCOR* and *CCNB3* transcripts were highly overexpressed in our patient’s tumor, as were the *HOX*-*A*, -*B*, and -*C* gene clusters (Table 2). Furthermore, analysis of our patient’s tumor (Table 1) matched the BCS-specific fingerprint of genes used in the Riggi_Ewing_sarcoma_progenitor signature, which can be used to distinguish between BCS and EWS.^5,10^ Most notable for this patient was the observation that multiple genes in the CDK4/6–RB pathway (Table 1; Fig 2C) were overexpressed, which made palbociclib, which has known pediatric dosing information, an attractive drug to use in this case.

**TABLE 2.**
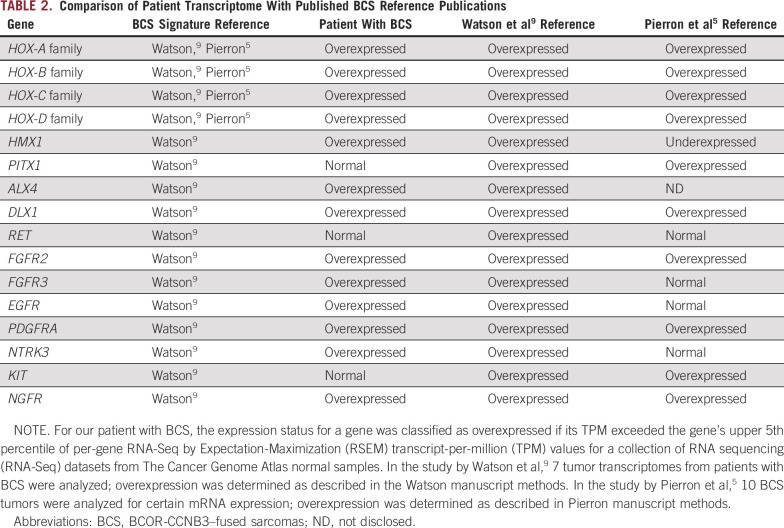
**Comparison of Patient Transcriptome With Published BCS Reference Publications**

Curiously, our patient was diagnosed at a very young age compared with the literature on BCS. In the several case series describing BCS, the median age of diagnosis is in the teenage years, with the youngest patient recorded at age 2.^5-8,11^ Again, because of the age of presentation, one could be concerned about an inherited cancer syndrome. Alfaro-Cervello et al^12^ published a case report of a congenital undifferentiated sarcoma with *BCOR*-*CCNB3* fusion possibly similar to our patient case. This congenital tumor also harbored a *SMARCB1*/*INI1* gene deletion common to malignant rhabdoid tumor, epithelioid sarcomas, and epithelioid malignant peripheral nerve sheath tumor that also, when found germline, is known to cause rhabdoid tumor predisposition syndrome.^12-16^ In the case reported by Alfaro-Cervello et al,^12^ *INI1* germline analysis was not performed. Our patient’s tumor had functionally intact *INI1*, which precludes an effective comparison. Our patient also harbored a germline heterozygous missense variant, *CDKN2B* N41D. It is unclear what role this germline *CDKN2B* N41D variant could play in sarcomagenesis, as cancer risks associated with *CDKN2A*/*B* gene variants include melanoma, pancreatic cancer, and astrocytomas.^17,18^ There is a recent short report from Jouenne et al^19^ that found an increased risk of soft tissue sarcoma development with germline loss of *CDKN2A*, though no data exist confirming this risk with *CDKN2B* variants. Additionally, little is known about this actual variant in *CDKN2B*. Sunita et al^20^ showed that the specific *CDKN2B* N41D variant, which encodes p15(INK4B), is unable to bind to the CDK6 protein, leading to loss of function of *CDKN2B*, which could lead to dysregulated control of S-phase entry. Though this variant’s contribution to tumorigenesis is intriguing, *CKDN2B* was normally expressed in our patient’s tumor, and there are no data suggesting that this impaired binding to CDK6 leads to mRNA overexpression along multiple levels of the CDK4/6 pathway.

Despite discovering alterations of several key regulators of the CDK4/6 pathway in this tumor, none have been proven to serve as clinical biomarker for sensitivity to CDK4/6 inhibitors.^21^ In a preclinical Ewing sarcoma orthotopic xenograft model with *CDKN2A* deletion, palbociclib was able to greatly suppress growth despite doxorubicin resistance of this model.^22^ In other sarcoma subtypes, palbociclib reduced tumor burden in murine preclinical models.^23-25^ Clinically, there is phase II evidence of palbociclib’s efficacy in adults with liposarcoma^26,27^ and leiomyosarcoma.^28^ Despite growing evidence in these sarcomas, there are no published data testing CDK4/6 inhibitors in BCS. Additionally, though phase I/II trials are underway, the only published response data for palbociclib in pediatrics is a case report of growing teratoma syndrome,^29^ thus making our use of this drug in a child novel.

To summarize, 3 independent observations supported consideration of therapeutic inhibition of the CDK4/6-RB1 pathway for this patient: (1) the presence of the *BCOR*-*CCNB3* gene fusion believed to drive entry into the cell cycle, (2) direct detection of an active CDK4/6-RB1 pathway, and (3) the presence of a germline *CDKN2B* variant. Using this information, our Precision Genomics team chose to place our patient with multiply-relapsed disease on palbociclib; the patient has now benefited from > 2 years of disease remission. The sustained complete response with palbociclib in our patient makes this case a novel and interesting application of palbociclib use and argues for additional research using CDK4/6 inhibitors in BCS.

## References

[1] Whang-Peng J Triche TJ Knutsen T et al Cytogenetic characterization of selected small round cell tumors of childhood Cancer Genet Cytogenet 21 185 208 1986 3004699 10.1016/0165-4608(86)90001-4

[2] Turc-Carel C Aurias A Mugneret F et al Chromosomes in Ewing’s sarcoma: I. An evaluation of 85 cases of remarkable consistency of t(11;22)(q24;q12) Cancer Genet Cytogenet 32 229 238 1988 3163261 10.1016/0165-4608(88)90285-3

[3] Ginsberg JP de Alava E Ladanyi M et al EWS-FLI1 and EWS-ERG gene fusions are associated with similar clinical phenotypes in Ewing’s sarcoma J Clin Oncol 17 1809 1814 1999 10561219 10.1200/JCO.1999.17.6.1809

[4] Shing DC McMullan DJ Roberts P et al FUS/ERG gene fusions in Ewing’s tumors Cancer Res 63 4568 4576 2003 12907633

[5] Pierron G Tirode F Lucchesi C et al A new subtype of bone sarcoma defined by BCOR-CCNB3 gene fusion Nat Genet 44 461 466 2012 22387997 10.1038/ng.1107

[6] Peters TL Kumar V Polikepahad S et al BCOR-CCNB3 fusions are frequent in undifferentiated sarcomas of male children Mod Pathol 28 575 586 2015 25360585 10.1038/modpathol.2014.139 PMC4385430

[7] Puls F Niblett A Marland G et al BCOR-CCNB3 (Ewing-like) sarcoma: A clinicopathologic analysis of 10 cases, in comparison with conventional Ewing sarcoma Am J Surg Pathol 38 1307 1318 2014 24805859 10.1097/PAS.0000000000000223

[8] Kao YC Owosho AA Sung YS et al: BCOR-CCNB3 fusion–positive sarcomas: A clinicopathologic and molecular analysis of 36 cases with comparison to morphologic spectrum and clinical behavior of other round cell sarcomas. Am J Surg Pathol, 42:604-615, 2018 29300189 10.1097/PAS.0000000000000965 PMC5893395

[9] Watson S Perrin V Guillemot D et al Transcriptomic definition of molecular subgroups of small round cell sarcomas J Pathol 245 29 40 2018 29431183 10.1002/path.5053

[10] Riggi N Suvà ML Suvà D et al EWS-FLI-1 expression triggers a Ewing’s sarcoma initiation program in primary human mesenchymal stem cells Cancer Res 68 2176 2185 2008 18381423 10.1158/0008-5472.CAN-07-1761

[11] Matsuyama A Shiba E Umekita Y et al Clinicopathologic diversity of undifferentiated sarcoma with BCOR-CCNB3 fusion: Analysis of 11 cases with a reappraisal of the utility of immunohistochemistry for BCOR and CCNB3 Am J Surg Pathol 41 1713 1721 2017 28877060 10.1097/PAS.0000000000000934

[12] Alfaro-Cervello C Andrade-Gamarra V Nieto G et al Congenital undifferentiated sarcoma associated to BCOR-CCNB3 gene fusion Pathol Res Pract 213 1435 1439 2017 28756981 10.1016/j.prp.2017.07.012

[13] Judkins AR Mauger J Ht A et al Immunohistochemical analysis of hSNF5/INI1 in pediatric CNS neoplasms Am J Surg Pathol 28 644 650 2004 15105654 10.1097/00000478-200405000-00013

[14] Modena P Lualdi E Facchinetti F et al SMARCB1/INI1 tumor suppressor gene is frequently inactivated in epithelioid sarcomas Cancer Res 65 4012 4019 2005 15899790 10.1158/0008-5472.CAN-04-3050

[15] Hornick JL Dal Cin P Fletcher CDM Loss of INI1 expression is characteristic of both conventional and proximal-type epithelioid sarcoma Am J Surg Pathol 33 542 550 2009 19033866 10.1097/PAS.0b013e3181882c54

[16] Sredni ST Tomita T Rhabdoid tumor predisposition syndrome Pediatr Dev Pathol 18 49 58 2015 25494491 10.2350/14-07-1531-MISC.1

[17] Chan AK Han SJ Choy W et al Familial melanoma-astrocytoma syndrome: Synchronous diffuse astrocytoma and pleomorphic xanthoastrocytoma in a patient with germline CDKN2A/B deletion and a significant family history Clin Neuropathol 36 213 221 2017 28699883 10.5414/NP301022 PMC5628627

[18] Campa D Pastore M Gentiluomo M et al Functional single nucleotide polymorphisms within the cyclin-dependent kinase inhibitor 2A/2B region affect pancreatic cancer risk Oncotarget 7 57011 57020 2016 27486979 10.18632/oncotarget.10935 PMC5302969

[19] Jouenne F Chauvot de Beauchene I Bollaert E et al Germline CDKN2A/P16INK4A mutations contribute to genetic determinism of sarcoma J Med Genet 54 607 612 2017 28592523 10.1136/jmedgenet-2016-104402

[20] Agarwal SK Mateo CM Marx SJ Rare germline mutations in cyclin-dependent kinase inhibitor genes in multiple endocrine neoplasia type 1 and related states J Clin Endocrinol Metab 94 1826 1834 2009 19141585 10.1210/jc.2008-2083 PMC2684477

[21] Knudsen ES Witkiewicz AK The strange case of CDK4/6 inhibitors: Mechanisms, resistance, and combination strategies Trends Cancer 3 39 55 2017 28303264 10.1016/j.trecan.2016.11.006 PMC5347397

[22] Murakami T Singh AS Kiyuna T et al Effective molecular targeting of CDK4/6 and IGF-1R in a rare FUS-ERG fusion CDKN2A-deletion doxorubicin-resistant Ewing’s sarcoma patient-derived orthotopic xenograft (PDOX) nude-mouse model Oncotarget 7 47556 47564 2016 27286459 10.18632/oncotarget.9879 PMC5216960

[23] Perez M Muñoz-Galván S Jiménez-García MP et al Efficacy of CDK4 inhibition against sarcomas depends on their levels of CDK4 and p16ink4 mRNA Oncotarget 6 40557 40574 2015 26528855 10.18632/oncotarget.5829 PMC4747352

[24] Vlenterie M Hillebrandt-Roeffen MH Schaars EW et al Targeting cyclin-dependent kinases in synovial sarcoma: Palbociclib as a potential treatment for synovial sarcoma patients Ann Surg Oncol 23 2745 2752 2016 27334220 10.1245/s10434-016-5341-x PMC4972869

[25] Böhm MJ Marienfeld R Jäger D et al Analysis of the CDK4/6 cell cycle pathway in leiomyosarcomas as a potential target for inhibition by palbociclib Sarcoma 2019 3914232 2019 30804704 10.1155/2019/3914232 PMC6360577

[26] Dickson MA Tap WD Keohan ML et al Phase II trial of the CDK4 inhibitor PD0332991 in patients with advanced CDK4-amplified well-differentiated or dedifferentiated liposarcoma J Clin Oncol 31 2024 2028 2013 23569312 10.1200/JCO.2012.46.5476 PMC3661937

[27] Dickson MA Schwartz GK Keohan ML et al Progression-free survival among patients with well-differentiated or dedifferentiated liposarcoma treated with CDK4 inhibitor palbociclib: A phase 2 clinical trial JAMA Oncol 2 937 940 2016 27124835 10.1001/jamaoncol.2016.0264 PMC4991028

[28] Elvin JA Gay LM Ort R et al Clinical benefit in response to palbociclib treatment in refractory uterine leiomyosarcomas with a common *CDKN2A* alteration Oncologist 22 416 421 2017 28283584 10.1634/theoncologist.2016-0310 PMC5388371

[29] Schultz KA Petronio J Bendel A et al: PD0332991 (palbociclib) for treatment of pediatric intracranial growing teratoma syndrome. Pediatr Blood Cancer 62:1072-1074, 2015 25417786 10.1002/pbc.25338

[30] Li B Dewey CN RSEM: Accurate transcript quantification from RNA-Seq data with or without a reference genome BMC Bioinformatics 12 323 2011 21816040 10.1186/1471-2105-12-323 PMC3163565

